# Searching with and against each other: Spatiotemporal coordination of visual search behavior in collaborative and competitive settings

**DOI:** 10.3758/s13414-018-01640-0

**Published:** 2018-12-28

**Authors:** Diederick C. Niehorster, Tim Cornelissen, Kenneth Holmqvist, Ignace Hooge

**Affiliations:** 1 Lund University Humanities Lab, Lund University, Box 201, SE-221 00 Lund, Sweden; 2 Department of Psychology, Lund University, Lund, Sweden; 3 Scene Grammar Lab, Department of Cognitive Psychology, Goethe University Frankfurt, Frankfurt, Germany; 4 Department of Psychology, Regensburg University, Regensburg, Germany; 5 Department of Psychology, Torun University, Toruń, Poland; 6 Faculty of Arts, Masaryk University, Brno, Czech Republic; 7 Experimental Psychology, Helmholtz Institute, Utrecht University, Utrecht, The Netherlands

**Keywords:** Visual search, Shared gaze, Collaboration, Competition, Eye movements

## Abstract

Although in real life people frequently perform visual search together, in lab experiments this social dimension is typically left out. Here, we investigate individual, collaborative and competitive visual search with visualization of search partners’ gaze. Participants were instructed to search a grid of Gabor patches while being eye tracked. For collaboration and competition, searchers were shown in real time at which element the paired searcher was looking. To promote collaboration or competition, points were rewarded or deducted for correct or incorrect answers. Early in collaboration trials, searchers rarely fixated the same elements. Reaction times of couples were roughly halved compared with individual search, although error rates did not increase. This indicates searchers formed an efficient collaboration strategy. Overlap, the proportion of dwells that landed on hexagons that the other searcher had already looked at, was lower than expected from simulated overlap of two searchers who are blind to the behavior of their partner. The proportion of overlapping dwells correlated positively with ratings of the quality of collaboration. During competition, overlap increased earlier in time, indicating that competitors divided space less efficiently. Analysis of the entropy of the dwell locations and scan paths revealed that in the competition condition, a less fixed looking pattern was exhibited than in the collaborate and individual search conditions. We conclude that participants can efficiently search together when provided only with information about their partner’s gaze position by dividing up the search space. Competing search exhibited more random gaze patterns, potentially reflecting increased interaction between searchers in this condition.

Much of what is known about cognition and behavior is derived from experiments that involve an individual observer performing experimental tasks by himself or herself. This approach allows for great control over experimental conditions and the stimuli presented to observers. However, this approach ignores the fact that humans spend significant parts of their day in interaction with others. Everyday interactions in such social settings involve many of the cognitive processes that these single-participant lab tasks purport to study, such as language processing (Pickering & Garrod, 2004) and decision-making (Campbell-Meiklejohn, Bach, Roepstorff, Dolan, & Frith, 2010; Cascio, Scholz, & Falk, 2015). Indeed, there is a growing body of research showing that performing basic lab tasks, such as antisaccades (Oliva, Niehorster, Jarodzka, & Holmqvist, 2017), inhibition of return paradigms (Skarratt, Cole, & Kuhn, 2012; Welsh et al., 2005), memory recall (Weldon & Bellinger, 1997) and go/no-go tasks (Dolk, Hommel, Prinz, & Liepelt, 2013) in the presence of others can yield results that differ substantially from those found when participants perform the task when alone in the experiment room. Such findings underscore that social context, such as the presence of others, is a factor in cognition.

Within the field of vision science, surprisingly few experiments are conducted where multiple participants interact, such as in collaboration or competition with another participant. A notable exception is Brennan, Chen, Dickinson, Neider, and Zelinsky (2008), who studied collaborative visual search. Brennan et al. (2008) had two collaborators perform a joint visual search task looking for an *O* among *Q*s. During the search, both observers were shown where in the display their partner was looking by means of a gaze marker (a ring) linked to the partner’s gaze location. Search performance in this shared gaze condition was compared with a condition in which searchers could communicate only verbally, a condition in which searchers could see where their partner was looking and verbally communicate, and a condition where searchers had access to neither source of information about their partner. As a control condition, the same search task was also performed by a separate group of single observers. Brennan et al. (2008) showed that when searchers were able to communicate, the coordination strategies they adopted were characterized by a division of the search display into two sides, with each searcher typically searching only one side and with little overlap between the areas covered by the searchers’ fixations. Interestingly, Brennan et al. (2008) found that reaction times of the team were shortest in the shared-gaze-only condition, where searchers were not able to talk with each other. Specifically, in the shared-gaze condition, the search slopes of the pair were shallower than in the no-communication condition, indicating that search was faster when collaborating.

While Brennan et al. (2008) reported that adding a second searcher who collaborated through shared gaze led to the team finding targets twice as fast, an experiment by Messmer, Leggett, Prince, and McCarley (2017) using a very similar search task and setup found a much more modest speedup as their paired searchers were only about 1.2–1.4 times faster than a single searcher. This indicates that in Messmer et al.’s (2017) experiment, seeing the other searcher’s gaze behavior through a gaze marker (a dot) caused individual searchers’ search efficiency to decrease. Messmer et al. (2017), however, did not report analyses of eye-movement data, making it unclear what led to the decrease in efficiency. Two other studies using a different search task (Neider, Chen, Dickinson, Brennan, & Zelinsky, 2010; Yamani, Neider, Kramer, & McCarley, 2017) also reported either no reduction in reaction time when two searchers were provided with shared gaze (Neider et al., 2010), or even an increase (Yamani et al., 2017), compared with a single individual searching the display. These latter two studies used a search task in which both searchers had to indicate having found the same target, instead of the trial ending after the first searcher found the target. This instruction induced searchers to precisely monitor their partner’s progress. Yamani et al. (2017) reported that this led to increased overlap in search area between searchers compared with when they were not provided access to the other’s search behavior through shared gaze.

The work by Brennan et al. (2008) has shown that collaborative visual search seems to be a relatively simple task to perform as a searcher, since the pairs of participants in their study naturally adopted a division-of-labor strategy even when they could not verbally communicate. Can the social visual search paradigm also be extended to the study of competitive situations, such as searching for scarce resources and (e-)sports? We argue that competitive settings are where this research paradigm can truly reveal its strength because interaction and feedback may play a much larger role in competition than collaboration for this task. For some forms of competition, namely those where there is little interaction between the individual competitors, it is calculable who will be the likely winner. For instance, in the case of competitive swimming, successful models exist to determine who will win a race based on a few biophysical parameters (Ingen Schenau & Cavanagh, 1990; Toussaint & Beek, 1992). The outcome of other forms of competition, such as most team sports, is, however, hard to predict based on individual performance. While the quality of the individual players is a factor in sports like ice hockey, which team wins the match is determined by the interaction between the two teams and the individual players in the teams, with factors such as executing unexpected strategies that surprise the opponent playing an important role (see also Eisenhardt & Brown, 1998, for business strategy; and Firestone & Warren, 2010, for escaping predators). Taking a hockey penalty shot as example, if the shooter would reliably execute the same play time after time, the goalie could easily predict the shooter’s actions and successfully block the shot. By analogy, successful behavior when performing competitive visual search may, for example, comprise searchers using the feedback of seeing where their competitor looks to try to adapt to their competitor’s strategy so that they can stay one search target ahead, while simultaneously trying to search less predictably to make it hard for their competitor to outmaneuver them. In contrast to collaborative visual search, where searching in a fixed predictable pattern is sufficient for successful collaboration, many competitive search strategies thus require continuous interaction between the searchers, and we may expect that the interaction will lead two searchers to forage less systematically through the search array. Importantly, it should be noted that division of space is not a winning strategy for competition as it would entail that each searcher wins only on a number of trials proportional to their relative search speed, as compared to their competitor’s speed.

Why study collaboration and competition using a visual search task? Saccadic search (Hooge & Erkelens, 1996, 1998, 1999; Over, Hooge, Vlaskamp, & Erkelens, 2007; Zelinsky, Rao, Hayhoe, & Ballard, 1997) is especially suited for studying collaboration and competition in a simple visual process for several reasons. First, visual search has been studied extensively, and therefore any study using this paradigm can build upon extensive knowledge regarding typical performance and behavior of single participants, as well as many available metrics (e.g., reaction time and accuracy) for quantifying performance. Second, shared gaze provides a communication channel that is spatially and temporally very precise. Specifically, shared gaze allows for near real-time transmission of spatial information. This arguably underlies the finding of previous research that shared gaze is a very efficient form of communicating when searching with two people. Verbal communication in comparison is spatially less precise (e.g., one can quickly convey an area by saying “left,” but probably not an exact coordinate) and incurs longer latencies for information transmission as speech unfolds more slowly over time. Third, as compared with recordings of verbal communication that require transcribing and scoring, or video observation methods which require subjective procedures to code events, eye-movement data can be quantified objectively, with little manual work. Last, if the information exchange between two partners occurs via visualized gaze positions, it can be measured online and in great spatial detail, potentially providing the researcher with insight into the process of collaboration rather than just the outcome (Jarodzka, Holmqvist, & Gruber, 2017), and also providing the experimenter with the opportunity to manipulate the interaction between partners experimentally as it unfolds.

In the current study, two participants searched either collaboratively or competitively in the same space while we monitored their eye movements. The eye-movement data were relayed and visualized to their partner in real time by means of a gaze marker to provide a shared gaze communication channel. The current study had four aims.

First, we aimed to replicate Brennan et al.’s (2008) finding that two collaborating searchers self-organize and minimize overlap in the elements looked at when only provided with shared gaze as a means of communication. Given the inconsistent results of the studies reviewed above with regard to the benefit of collaboration through shared gaze for team search speed, we also aim to replicate the doubling of search speed of the pair compared with a single searcher that Brennan et al. (2008) reported.

Second, we investigated what strategy is adopted by competing searchers, and how this differs from collaborating searchers. Does shared gaze enable faster search for two searchers also when searchers are instructed to compete, rather than collaborate? Do competing searchers still divide the search space? Do competing searchers show more random and unpredictable scanning behavior than collaborating searchers do? While a participant may have a higher chance to win by scanning faster than their competitor, this is not the only way to win. Participants may interact since they are provided with information about where their competitor looks. For example, if an opponent looks in a highly predictable pattern, one could win by looking one element ahead of the opponent. In turn, this means the opponent could decrease their chance of losing by being *less* predictable. In the competition condition, we are thus potentially investigating a complicated feedback system. This entails that many intercompetitor interactions and their associated gaze patterns are possible. To develop a first insight into what behavioral strategies competing searchers adopt, we introduce analysis methods using entropy measures, which quantify the amount of disorder (or order) in a pattern. Using these measures, we examine whether the scanning behavior of the searchers is more random over trials and thereby less predictable in the competition condition than in the collaboration and individual conditions.

Third, we aimed to provide a more complete picture of collaborative visual search supported by shared gaze. We therefore investigated how it unfolds over time. Furthermore, during pilot experiments, the current authors experienced pressure to perform faster than their partner in the collaborative condition that may lead to a certain amount of competitive behavior being exhibited in the collaborative condition. As such, the extent to which searchers’ behavior truly reflects collaboration when the instruction to collaborate is given can be better appreciated in contrast to a condition in which searchers are explicitly instructed to compete.

Last, we investigated by means of a questionnaire how participants experienced collaborating or competing with another participant while being provided with a real-time visualization of where the paired searcher is looking. We furthermore investigated how participants’ ratings of the extent to which their partner collaborated or competed related to the observed search behavior.

## Method

### Participants

Thirty-six participants (22 female, mean age = 29 years, *SD* = 7 years) took part in this study, and were divided into eight experimental sessions that consisted of either four or six participants at a time. Only participants from whom it was possible to obtain an accurate calibration of the eye tracker, whose gaze signal did not exhibit high noise, and who were able to identify the target in a set of practice stimuli were selected to take part in this study. This ensured that the gaze visualization presented to each partnered searcher was of high quality (see Data Quality section). Each participant was briefly tested for these prerequisites before being allowed to take part in the study. On the basis of these criteria, four further potential participants were sent away before partaking. From the remaining participants, two (one pair) had to be excluded on the basis of data quality issues. The participants provided informed consent.

### Apparatus

All recordings were made in a room dubbed the “Digital Classroom” at the Lund University Humanities Lab. This lab is equipped with 25 RED-m eye-tracking devices from SensoMotoric Instruments (SMI) that are connected together via a high-bandwidth wired network by a Cisco SG500-52 switch. Each individual eye-tracker setup included a Dell laptop used in clamshell mode and a standard keyboard and mouse. Stimuli were presented on 22-inch Dell P2210 screens with a resolution of 1680 × 1050 pixels, 473 × 296 mm dimensions, and a refresh rate of 60 Hz. To maximize data quality (cf. Hessels, Cornelissen, Kemner, & Hooge, 2015; Niehorster, Cornelissen, Holmqvist, Hooge, & Hessels, 2017) and to fix viewing distance, participants were seated with their heads in a chin and forehead rest, 65 cm away from the screen. Gaze data were recorded from both eyes individually, at a sampling rate of 120 Hz. Stimuli were presented using MATLAB, combined with the Psychophysics Toolbox (Brainard, 1997; Pelli, 1997). Because between four and six participants along with two experimenters were present in the same room during the experimental sessions, a number of measures were taken to ensure that walking around, whispers, and the sound of buttons being pressed would minimally disturb participants. A 2-m high cardboard divider was placed around the back, left, and right of each individual participant’s table, and tables were placed to face the center of the room, maximally spaced from one another. Furthermore, participants wore closed over-ear headphones (Sennheiser HD 201, Wedemark, Germany) throughout the experimental sessions to block out as much sound from the room as possible. No sound was presented through the headphones.

### Stimuli

**Fig. 1:**
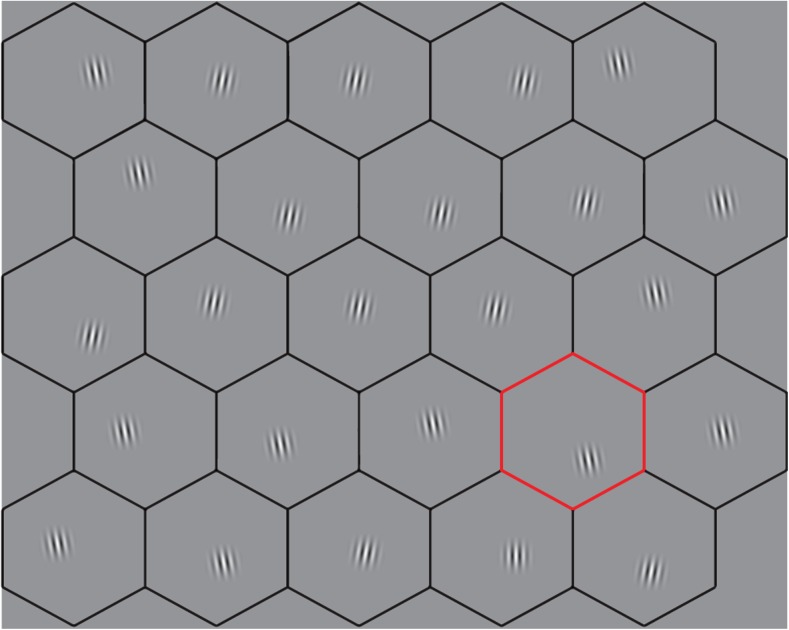
Illustration of the search stimulus used in this study. For illustration purposes, contrast modulation in the Gabor patches was increased 1.6 times to the full luminance range, the frequency of the sinusoid carrier decreased threefold, and the sigma of the Gaussian envelop enlarged by two-thirds, compared with the stimuli used in the experiment. The red outlined hexagon is the gaze marker indicating where the search partner is currently looking. The target is located in the bottom row, second from the right. (Color figure online)

In each trial a unique search display was shown to each participant or pair of participants. Each search display measured 1040 × 1040 pixels in size and was presented centered on the screen, spanning 25.8° × 25.8° at 65-cm viewing distance. The horizontal extremities of the screen were not used because eye-tracker data quality decreases in these areas (Holmqvist & Andersson, 2017, p. 173), posing potential problems for both gaze data analysis and gaze data visualization. Displays had a gray background (128 on 8-bit grayscale). Search elements were 25 Gabor patches in each stimulus, distributed across a 5 × 5 hexagonal grid with a distance of 4.6° between the centers of adjacent hexagons. To place elements in each hexagon, a horizontal and a vertical shift were added individually from each hexagon center, with amplitudes of both shifts ranging randomly from 0° to a maximum of 0.805°. The borders of the hexagons defining the hexagonal grid were visible as black lines throughout each trial. The diameter of the hexagon (from straight edge to opposite straight edge) spanned 4.6°. All Gabor patches had a carrier frequency of 12 cycles per degree, and the standard deviation of the overlaid Gaussian was set to 0.15°, resulting in a Gabor of roughly 0.7° radius. The amplitude of the underlying sinusoidal carrier was 80 gray values centered around the background color. The Gabor patches were pretested by the authors to ensure that they could not be identified peripherally when fixating the element in an adjacent hexagon. All distractors were rotated 10 degrees clockwise or counterclockwise from vertical. The target was a single Gabor patch with a vertical orientation and was present in all trials (see Fig. 1 for an example). Target location was randomized from stimulus to stimulus, but a target never appeared in the middle hexagon, which is where participants were instructed to look at stimulus onset. Gabor patch properties and placement were chosen to ease localization in the visual periphery, but make identification of the target difficult without foveation.

During trials in the paired conditions, the gaze location of the searcher’s partner was represented by outlining in red the hexagon in which the partner was currently looking, according to the partner’s eye tracker (see Fig. 1). Using an outlined hexagon as a gaze marker, rather than Brennan et al.’s (2008) dot indicating gaze position, was done for two reasons. First, this method minimized jumpy movement of the gaze marker on screen due to a relatively low precision of the eye trackers used. Secondly, outlining an area allows inspection of the same hexagon by two searchers simultaneously, something that is not possible when a search element is obscured by a gaze marker. The hexagon where the partner looks was determined by calculating for each streamed gaze sample that arrived in the last 30 ms (3–4 samples) which hexagon it landed in. The hexagon that most samples landed in was highlighted. If all samples indicated a different hexagon, it was assumed that a saccade was in progress, and the hexagon that the latest incoming sample fell into was outlined. Information about which hexagon was highlighted each frame was saved to file to allow post hoc reconstruction of what had been presented to each participant during the trial.

### Design

Each participant completed three conditions: an *individual*, a *collaborative*, and a *competitive* condition. The individual condition served as a baseline and consisted of all searchers in the session starting the search task simultaneously, and then completing it at their own pace, without any kind of information about the others’ search behavior or performance. The individual condition was always run first. Participants then completed the collaborative and competitive conditions in counterbalanced order.

In the individual condition, participants were instructed to search for a target, which was present on every trial, as fast and as accurately as possible. After giving a speeded response by button press, accuracy was judged by removing the search elements but leaving the hexagonal grid on the screen, and having participants click the hexagon where they had found the target.

In the collaborative condition, participants were informed that they were to search for the same target as they had in the individual condition, but that they would now be paired with another searcher in the room, picked at random. It was made clear that the hexagon where the other searcher is looking would be outlined in red. Participants were instructed to find the target as fast and accurately as possible, but this time to do so *together* with the person they were paired with. Whichever of the two partners that had found the target was to respond the same way as in the individual condition. To enhance collaboration, participants were *both* rewarded 300 points for correct responses and both were penalized 600 points for incorrect responses (cf. Brennan et al., 2008, who provided monetary reward).

In the competitive condition, participants were given the same information about being paired with another searcher, with the exception that they were told to *compete* against the other searcher by finding targets faster than their opponent. Whichever of the two search partners that had found the target was to respond the same way as in the individual condition, but only the searcher who responded earned 300 points for a correct response, or was penalized 600 points for an incorrect response. In the individual condition, the same amount of points was given or taken away for correct and incorrect responses.

Feedback about correct or incorrect responses, and the current score was given after each trial, in all conditions. Although searchers were told that their partner was chosen randomly from all participants in the room for each condition, the same two searchers were in fact paired up for the collaborative and competitive conditions for comparability during analysis.

### Data streaming

With the available infrastructure combined with custom-built software, we were able to stream gaze data from computer to computer at submillisecond latency at the utilized sampling frequency of 120 Hz. This allowed us to synchronize stimulus onset between searchers with a precision of one screen-refresh interval, and ensured that the gaze information used to indicate where a paired searcher looks was current. For a more specific description of the setup, latency testing methods, and an earlier version of the software used in this study, see Nyström, Niehorster, Cornelissen, and Garde (2016). Current versions of the C++ software libraries and the MATLAB wrapper used in this study are available from https://github.com/dcnieho/UDPMultiCast. During the experiment, a separate computer kept track of the state of each eye-tracker station and pair of participants, signaling to each of them when to start calibrating, which stimulus to show, and when to start showing that stimulus—in essence, controlling the progress of the experiment.

### Procedure

Participants were welcomed to the lab and asked to sit down in front of one of the screens. Upon entering, participants were instructed not to speak to one another anymore. The group was then verbally informed about the calibration procedure and about the search task in general and only informed about shared gaze, collaboration or competition right before the start of the respective conditions, after completing the individual search condition. The setup procedure for each condition was as follows. Once all participants were correctly positioned in the chin rests, calibration was started simultaneously. After all participants had successfully completed a 5-point calibration and a 4-point validation procedure (average validation error 0.8°), written instructions for the current condition were presented. Once all participants indicated by button press that they had read and understood the instructions, the first trial of the condition began for all searchers.

**Fig. 2:**
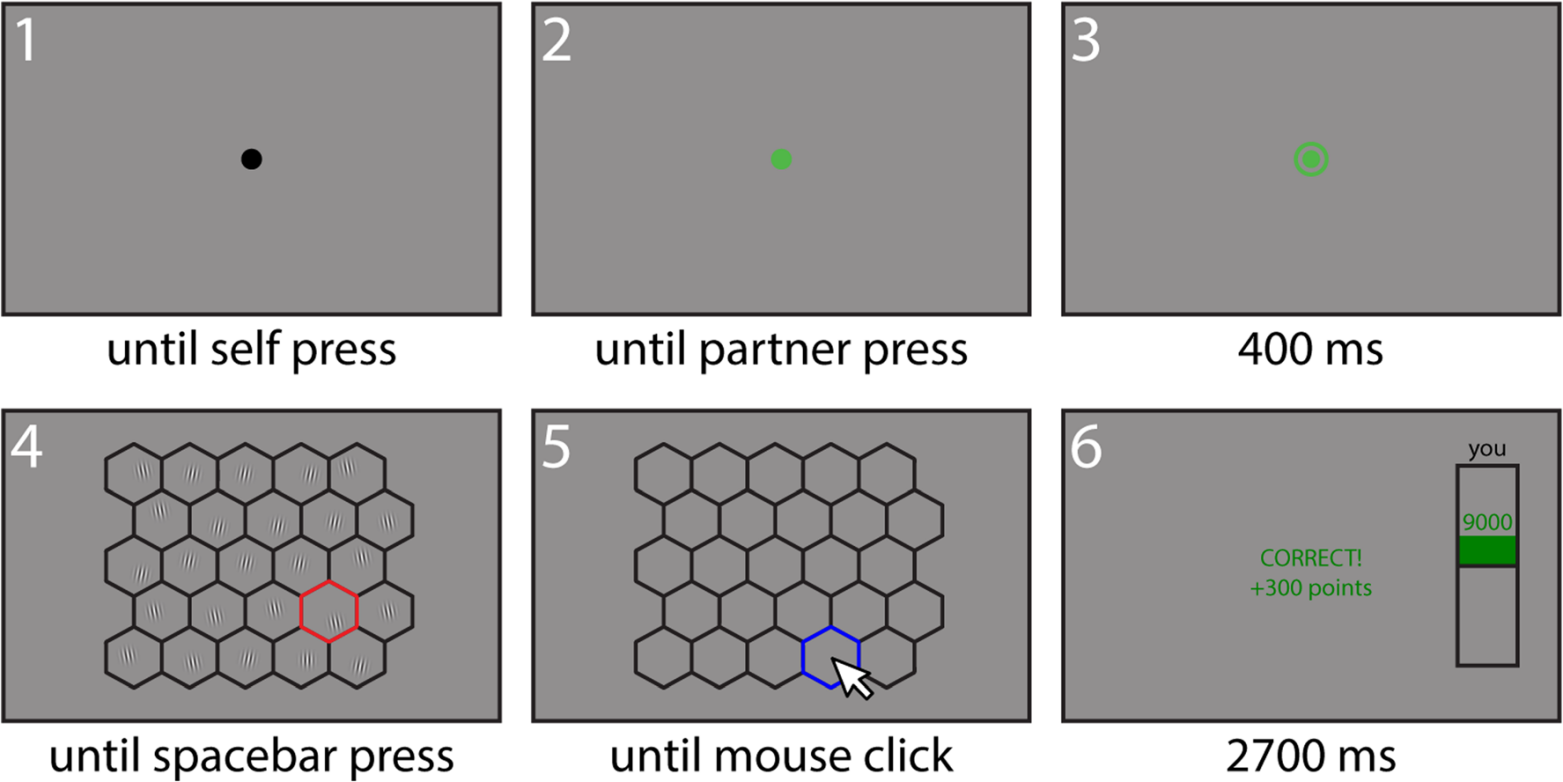
Trial sequence. Each trial consisted of six phases. Some phases had a variable length, which depended on an action of the participant or their partner. For these panels (1, 2, 4 and 5), the required action is noted below the panel. Other phases had a fixed duration. The duration of these phases (3 and 6) is denoted below the respective panels. (Color figure online)

See Fig. 2 for an overview of the timeline of a trial. Each trial started with a black fixation dot that was presented until key press. Once the key was pressed, this dot would turn green to indicate that the button press was registered. In the collaboration and competition conditions, both searchers were required to press a button to indicate that they were ready to start a trial. In this situation, it was possible that one searcher had already pressed the button, but had to wait for the other searcher to be ready and the trial to start. The fixation dot would therefore turn green to inform the participant that the button press was registered. Then, once *both* searchers had indicated they were ready, it would be followed by a green circle around the fixation dot, which indicated that the next search stimulus was about to be displayed to avoid surprise. In the individual condition, the circle would appear 300 ms after the key press and the dot turning green. In all conditions, the search array was presented 400 ms after the appearance of the circle.

Once a searcher ended the trial by pressing the space bar to indicate they had found the target, the search array was immediately removed from the screen (for both searchers if during the paired conditions). For the searcher who had responded, the stimulus was then replaced with only the lines of the hexagonal grid, but not the search elements. This searcher was now prompted to indicate the hexagon in which they found the target via mouse click. In the paired conditions, the searcher who had not responded was only presented with a gray screen. Upon clicking a hexagon, the searchers were given feedback about the correctness of the click and the change of their score. In the individual condition and the collaborative condition, this was done by presenting one score bar and the change in total score. For the competitive condition, two score bars were presented to indicate the current scores—one bar was labeled “you” and the other bar was labeled “other.” Each condition started with a series of 10 practice trials, which were not included for analysis and after which scores were reset to zero. Then, data were collected for another 100 trials for each condition.

At the conclusion of the experiment, participants were asked to fill out a brief questionnaire designed to measure several aspects of how they experienced the search task. Specifically, participants were asked to rate their agreement with eight statements on a Likert scale from 1 (*disagree*) to 10 (*agree*). Four of these statements were items about the extent to which they themselves collaborated or competed with the other searcher when asked to do so, and the extent to which the other searcher collaborated or competed with them. The other four statements gauged whether being provided with the marker showing where the other was currently looking was useful, and whether it was distracting during the collaborate and compete conditions. For each of these eight items, space was provided for optional additional comments.

### Data quality

For the purpose of this study, gaze data must be of sufficient quality for both off-line and online area-of-interest (AOI) analyses. Regarding off-line AOI analysis, this question is no different for this study than for many other eye-tracking studies (e.g., Hessels, Kemner, van den Boomen, & Hooge, 2016a; Orquin, Ashby, & Clarke, 2016; Orquin & Holmqvist, 2017). For the online AOI analysis used for gaze visualization, data has to be of sufficient quality to accurately represent at what hexagon a searcher is currently looking. Data of low precision could potentially cause a constantly shifting hexagon highlight when a searcher is really fixating a single hexagon, whereas low-accuracy data could systematically represent gaze as being in a different hexagon than it really is. Accepting only low enough validation errors ensures sufficient accuracy for both usages of the gaze data. Precision in terms of sample-to-sample RMS distance of the gaze data, averaged across participants, was 0.30°, and in terms of mean standard deviation, 0.42°. These values did not differ between conditions. To obtain a practical indication of whether these values constitute sufficient data quality for gaze visualization by means of hexagon highlighting, we analyzed the representation of the gaze signal on the monitor of the searcher to which it was streamed. For this analysis, all hexagon highlights that lasted for less than 50 ms, visible to the participants as a flickering of the highlighted hexagon, were taken to result from measurement error. This yielded on average less than 3% of stimulus frames per participant that were flagged as having visualized measurement error for both paired conditions. Note that this is an overestimation of measurement error, since saccades and blinks can result in brief outlining of hexagons, too, but this flicker would be due to real eye movements.

### Analysis

As indicators of search performance, we measured reaction times (RT) and proportions of trials where the correct response was given (defined as clicking the hexagon where the target was presented). Then we took several measures of search behavior. As a first analysis step, fixations were detected using the raw gaze signals from both eyes using the I2MC algorithm (Hessels, Niehorster, Kemner, & Hooge, 2016b). The I2MC algorithm is a clustering-based algorithm that was chosen because it was designed to deliver consistent fixation detection output across a wide range of noise levels and amounts of data loss, thereby minimizing the effect of differences in data quality between participants or trials on our results. After fixation detection, fixations were automatically assigned to a hexagon with custom-made software. For an indication of search speed (in addition to reaction times), we calculated the dwell rate and fixation duration. Dwell rate was defined as the number of hexagons visited per second. To further characterize search behavior, we also calculated the saccade size, and the size of transitions. A transition is defined as a saccade that carried gaze from one hexagon to another, and thus does not include saccades within a hexagon.

All data that are not time series are visualized using boxplots. Boxplots are constructed with a central line mark indicating the median value and with a box extending from the 25th (*q*_1_) to the 75th (*q*_3_) percentile of the data. Whiskers are drawn for data up to *q*_3_ + 1.5*iqr* and *q*_1_ − 1.5*iqr*, respectively, where *iqr* = *q*_3_ − *q*_1_. Any values outside these limits are shown individually. Since data in this article are reported in the form of medians and boxplots, nonparametric tests matching these metrics were used. Note that this likely decreased statistical power. Statistical analysis of medians comprised submitting medians per participant to Kruskal–Wallis tests, followed by post hoc Wilcoxon signed-rank tests between conditions where needed. For the Kruskal–Wallis test, *η*^2^ was calculating by first transforming the Kruskal–Wallis H statistic to an *F* value using the MATLAB function *finv*, and then computing the effect size from this value according to Lakens (2013). Following Grissom and Kim (2012), effect sizes for the Wilcoxon signed-rank test were indicated using the measure *PS*_*dep*_ = *n*_+_/*N*, where *n*_+_ is the number of positive difference scores out of a total of *N*.

To investigate whether or not participants were using a strategy that involved dividing the search task spatially, we calculated cumulative overlap over time. Overlap was calculated per participant and was defined as the number of hexagons on the participant’s screen that had both been highlighted as fixated by their partner and inspected by the searcher viewing this screen. For this measure, short marker presentations (hexagons highlighted for 50 ms or less, see Data Quality section) were ignored. For this measure, overlap would be 1 at the beginning of each trial and would start to increase as soon as both searchers inspected the same hexagons, up to a maximum of 1 if both searchers had looked at all hexagons in the grid. A strategy that involves successful division of labor should, on average, yield little overlap until a target is found. More specifically, if searchers divided space when collaborating, but not or less so when competing, then overlap is expected to increase later and possibly more slowly when searchers collaborate than when searchers compete.

To characterize scanning behavior, we further analyzed how dwells were distributed across the search array and to what extent participants’ scanning behavior followed a fixed looking pattern. To this end, we computed two entropy measures. First, we computed the Shannon entropy of the distribution of dwell locations of a searcher across the search array:

$$ {H}_d(X)=-\sum \limits_{i=1}^np\left({x}_i\right){\log}_2p\left({x}_i\right), $$

where *H*_*d*_ is the entropy in bits, *p*(*x*_*i*_) is the proportion of dwells on each item in the search array across all trials in a condition, and *n* is the number of search items. The entropy of the dwell distribution indicates the extent to which dwells are equally distributed (disordered) across the search array (maximum entropy) or biased to fall more on some of the elements in the search array (ordered, indicated by lower entropy). For a more in-depth introduction to entropy, see Hooge and Camps, 2013. Second, we examined whether searchers exhibited looking patterns that were random and unpredictable (high entropy) across trials or fixed and predictable (low entropy), regardless of what the looking pattern was. This was assessed using a conditional information metric (Allsop & Gray, 2014; see also Ellis & Stark, 1986) to calculate the entropy of the transitions between search elements made by each searcher:

$$ {H}_t(X)=-\sum \limits_{i=1}^np\left({x}_i\right)\left[\sum \limits_{j=1}^np\left({x}_{j\mid i}\right){\log}_2p\left({x}_{j\mid i}\right)\right],i\ne j, $$

where *p*(*x*_*j* ∣ *i*_) is the number of transitions from search item *i* to search item *j* across all trials in a condition as a proportion of all transitions originating from item *i*. These entropy measures provide sensitive quantities that allow us to compare searcher behavior across conditions in a quantitative manner, going beyond ad hoc measures or quantitative surveys of scan patterns and heat maps.

To obtain a baseline for a pair’s search performance and provide insight into how the availability of shared gaze changed search behavior, we modeled the joint performance of two noninteracting searchers. This baseline will be referred to as the blind condition in this manuscript, as it reflects expected behavior of a pair of participants who were blind to each other’s visual exploration behavior. This baseline was constructed using data from the individual condition for each pair. As each participant saw unique search arrays, blind simultaneous search performance was estimated by using matched trials where the target was at the same location in the search array and for each such trial setting the response and RT to that of the faster searcher. On average, this procedure yielded 71 such artificial trials per pair of participants (range: 52–80).

## Results

### Search performance

**Fig. 3:**
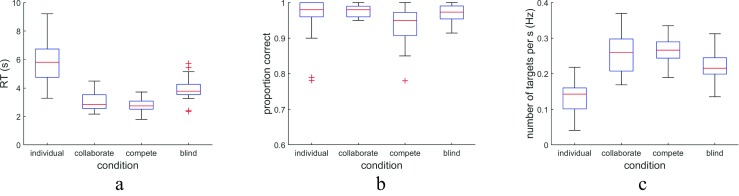
Reaction time, proportion correct, and number of targets found per second, in the three search conditions, along with simulated blind search. In the individual condition, one participant had a median reaction time of 14 s (not shown)

How did the searchers perform individually and when collaborating or competing? Figure 3a shows mean reaction times (RT) across all searchers for the three search conditions and the blind baseline. The median RT when searching individually was 5.80 s, indicating that searchers had to inspect each element individually. The median reaction time in the collaborate condition (2.84 s) was roughly half of that in the individual condition, indicating that two collaborating participants found the target twice as fast compared with a single participant searching alone. At this duration of collaborative trials, there likely is sufficient time within each trial for searchers to note where their partner is fixating and coordinate their own scanning behavior. Targets were detected a further 92 ms faster in the compete condition than in the collaborate condition (*Z* = 2.11, *p* = .035, *PS*_*dep*_= 0.59).

Few errors were made in all conditions (see Fig. 3b). In both the individual and the collaborate conditions, the median error rate was 2.0%. The median error rate increased to 5.0% in the compete condition (*Z* = 3.12, *p* = .0018, *PS*_*dep*_ = 0.76). The slight increase in search speed together with the increased error rate suggests that a speed–accuracy trade-off occurred. However, an analysis of the number of targets found per second (see Fig. 3c) revealed no difference between the collaborate and compete conditions (median: 0.26 Hz vs. 0.27 Hz, *Z* = 0.45, *p* = .65, *PS*_*dep*_= 0.53), indicating that search at this different point on the speed–accuracy trade-off was equally effective. In both the collaborate and compete conditions, approximately 1.8 times more targets were found per second than in the individual conditions (median: 0.14Hz). We furthermore analyzed whether searchers overlooked the target (defined as looking at two or more hexagons after first fixating the hexagon containing the target) more often when competing or collaborating than when searching alone. No differences in overlooking were found between the conditions, *H*(2) = 1.44, *p* = .49, *η*^2^= 0.01.

A comparison with the blind search condition, which estimated the joint performance of two noninteracting searchers, revealed that providing searchers with shared gaze yields 943 ms shorter median search times in the collaborate condition (*Z* = 3.05, *p* = .0023, *PS*_*dep*_ = 0.76), and no difference in median error rate (2.0% vs. 2.7%, *Z* = 0.47, *p* = .64, *PS*_*dep*_ = 0.53). This indicates that the speed-up made possible by shared gaze does not induce a speed–accuracy trade-off compared with searching without information about where a paired searcher is looking. Compared with the blind condition, median search time in the compete condition was 1,035 ms lower (*Z* = 3.62, *p* = .00029, *PS*_*dep*_ = 1.00), but at the cost of a significantly higher median error rate (5.0% vs. 2.7%, *Z* = 2.36, *p* = .019, *PS*_*dep*_ = 0.71). Furthermore, while the number of targets found per second was 1.5 times higher in the blind condition than in the individual condition, it was significantly lower (median: 0.22 Hz) than in the collaborate and compete conditions (*Z* = 2.44, *p* = .015, *PS*_*dep*_ = 0.82, and *Z* = 2.86, *p* = .0042, *PS*_*dep*_ = 0.88, respectively). This indicates that searching using shared gaze is more effective than searching in the same array without information about where the search partner looks.

### Eye-movement parameters

**Fig. 4:**
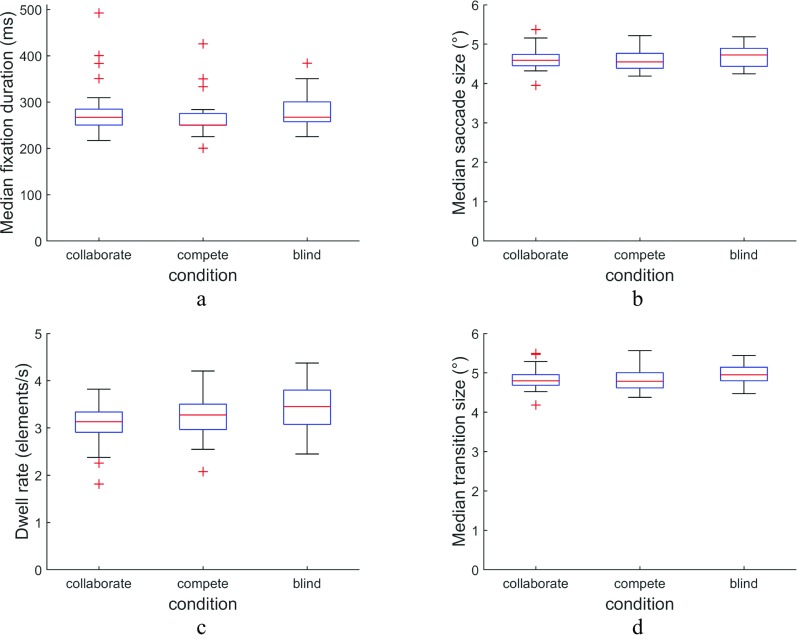
Eye-movement parameters per condition. Presented are (**a**) fixation duration, (**b**) saccade amplitude, (**c**) dwell rate (number of elements inspected per second), and (**d**) transition size (size of saccades that moved the gaze to another element)

Did the instruction to collaborate or compete lead to different eye-movement parameters? Figure 4a plots the median fixation duration of individual searchers in the three conditions. There was a statistically significant difference in median fixation duration between the conditions, *H*(2) = 6.95, *p* = .031, *η*^2^ = 0.067. Separate Wilcoxon signed-rank tests revealed that while the median fixation duration did not differ between the collaborate and blind conditions (*Z* = 1.27, *p* = .61, *PS*_*dep*_ = 0.53), it was shorter in the compete condition than in the collaborate and blind conditions (*Z* = 3.67, *p* = .0007, *PS*_*dep*_ = 0.85, and *Z* = 4.49, *p* < .0001, *PS*_*dep*_= 0.88, respectively). This suggests that in the compete condition, the speed-up in search time reported above may have been due to a reduction in fixation durations. There were no statistically significant differences in the median saccade sizes (see Fig. 4b) for the three conditions, *H*(2) = 4.84, *p* = .089, *η*^2^= 0.047.

Does the change in fixation duration translate into a change in the rate at which elements of the search display are inspected? Figures 4c–d plot the dwell rate (number of elements inspected per second) by individual searchers in the three conditions, and the median transition size (size of saccades that moved the gaze to another element). There was a statistically significant difference in dwell rate between the conditions, *H*(2) = 8.00, *p* = .018, *η*^2^ = 0.077. Separate Wilcoxon signed-rank tests revealed that the dwell rate was higher in the compete and blind conditions than in the collaborate condition (*Z* = 2.93, *p* = .010, *PS*_*dep*_ = 0.71, and *Z* = 3.55, *p* = .0012, *PS*_*dep*_ = 0.74, respectively), but that the dwell rate in the compete and blind conditions was not significantly different (*Z* = 1.77, *p* = .23, *PS*_*dep*_ = 0.59). Indeed, the shorter fixation duration in the compete than in the collaborate condition came along with a higher dwell rate. The higher dwell rate in the blind than in the collaborate condition despite equal fixation durations may suggest that the difference in dwell rates between these conditions is due to searchers making less fixations on an individual element before moving on to the next. There were no statistically significant differences in the median transition sizes for the three conditions, *H*(2) = 4.79, *p* = 0.091, *η*^2^ = 0.047.

### Spatiotemporal coordination between searchers

**Fig. 5:**
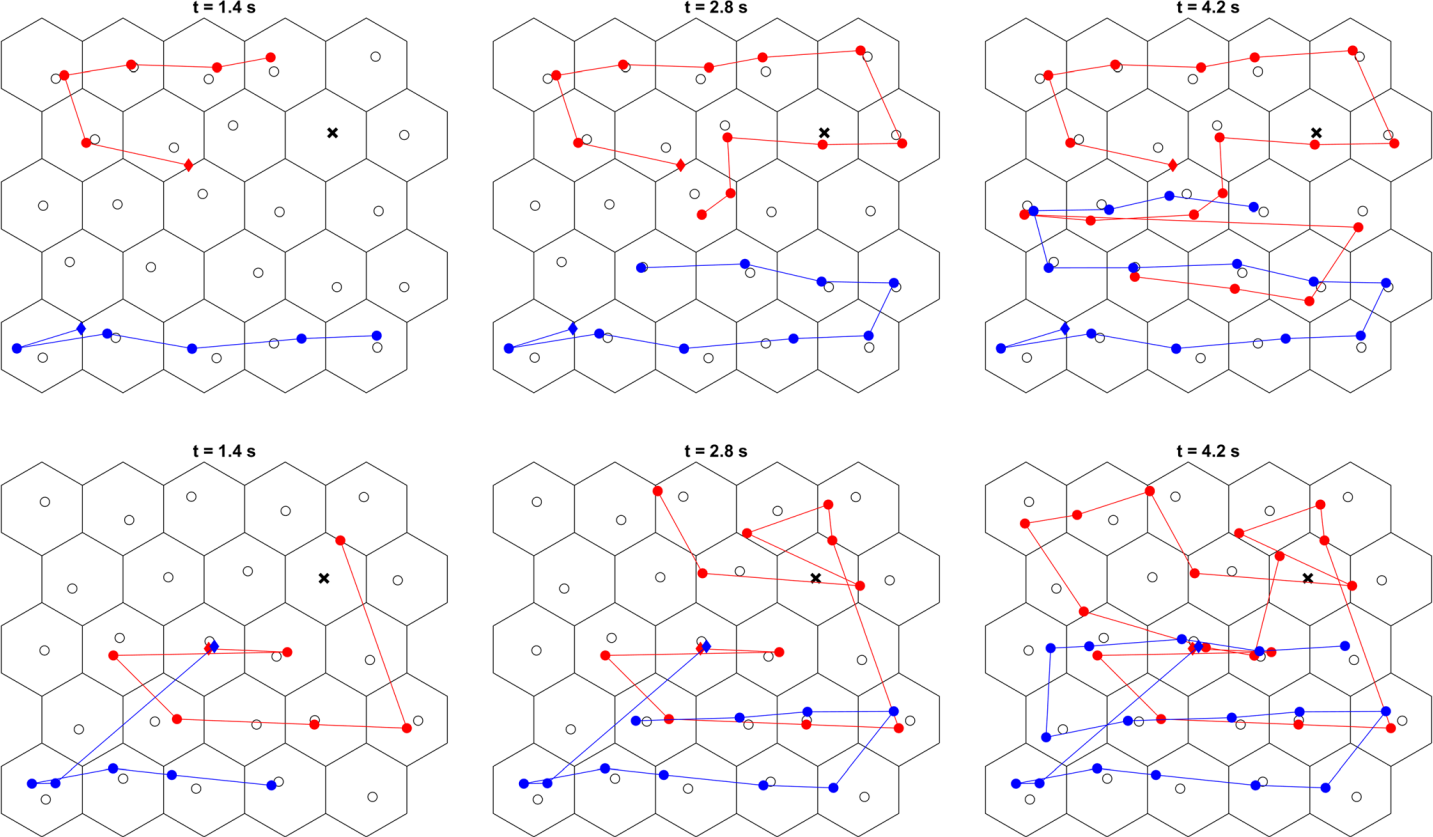
Scan paths for two participants in example collaborate and compete trials, at three time points. (Top row) collaborate condition; (bottom row) compete condition. These example trials come from the same pair and for both panels, a trial was chosen were the red searcher overlooked the target to provide sufficiently long search times for illustration purposes. Coloring (red: Searcher 1, blue: Searcher 2) is stable across all panels. This pair completed the collaborate condition first. The location of the first fixation is indicated by a diamond. The locations of distractor elements are indicated by open circles, and the location of the target by the bold *x*. (Color figure online)

How was search coordinated? Figure 5 contains one example trial from the collaborate and compete conditions each from the same pair, displaying the scan paths of the searchers. To provide insight into how collaborative and competitive search evolves during a trial, these plots are made at three time points in the trial. In the collaborate condition, we see that the participants started at different locations in the search array and initially searched near their starting point. They methodically moved to neighboring grid cells with each transition. Dwells in “each other’s” sides of the display only occurred once most of the elements on the participants’ “own” sides had been looked at. In contrast, in the compete condition, which was completed after the collaborate condition by this pair, we see that while the blue searcher maintained the same viewing behavior as in the collaborate condition, the red searcher no longer appears to start at the opposite side of the display, searched the display without a clearly visible systematic search strategy, and did not attempt to avoid looking where the other searcher has looked.

**Fig. 6:**
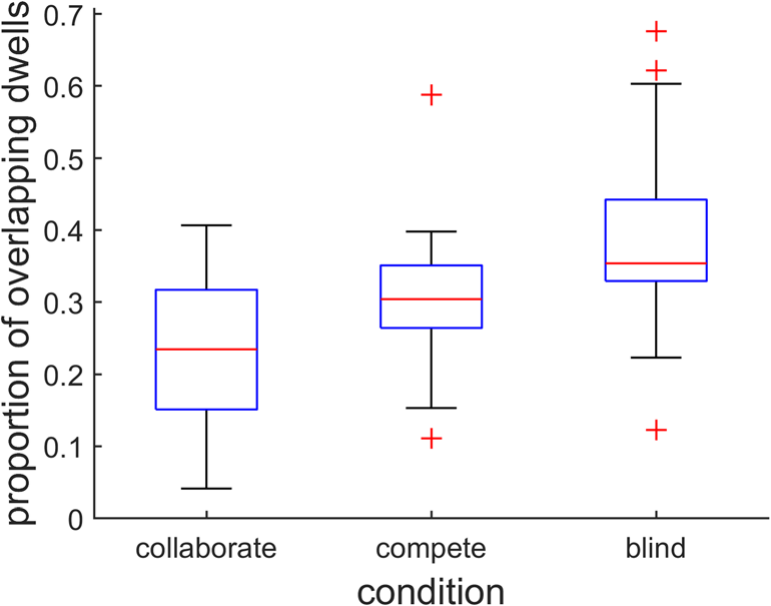
Overlap in looked at hexagons. Boxplot of the proportion of dwells that landed on hexagons that had already been looked at by the paired participant, for the collaborate and compete conditions, along with simulated blind search

To investigate the spatial distribution of dwells for all pairs, we calculated the proportion of dwells made by the participants that landed on hexagons that had already been looked at by their partners (see Fig. 6). This showed that significantly more dwells overlapped in the compete than in the collaborate condition (*Z* = 3.63, *p* = .00028, *PS*_*dep*_= 0.79). Furthermore, overlap for simulated blind search was significantly higher than in the compete condition (*Z* = 3.50, *p* = .00047, *PS*_*dep*_= 0.71).

**Fig. 7:**
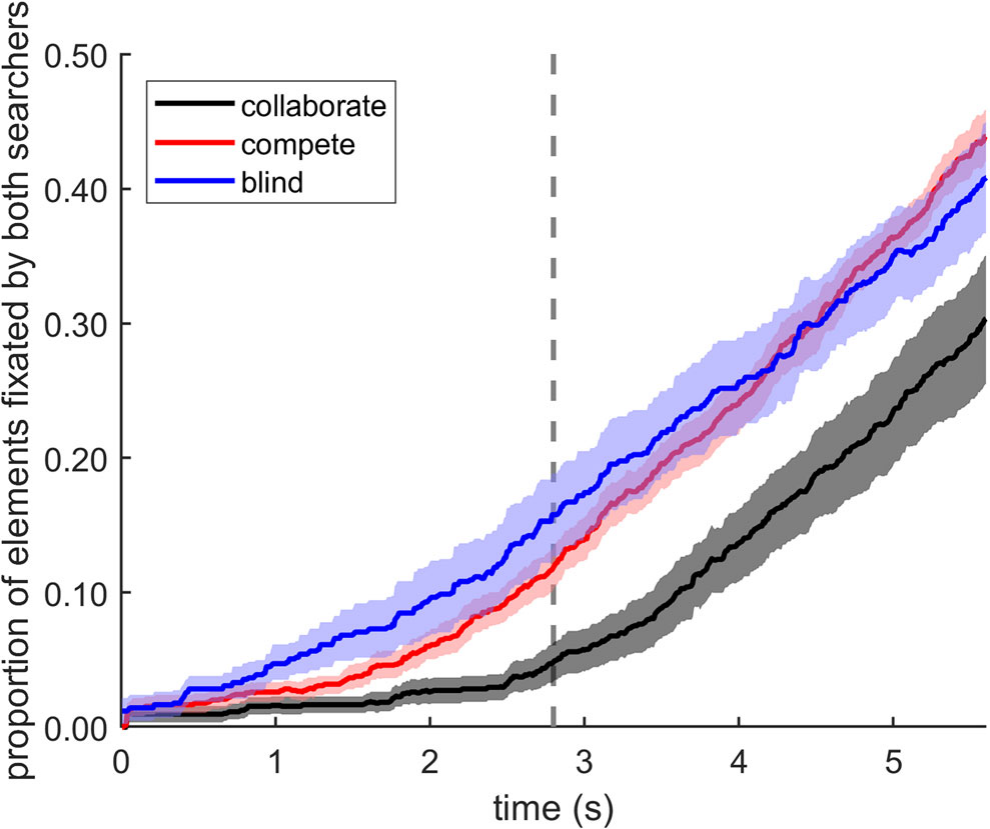
Overlap. Cumulative proportion of elements looked at by both searchers, as a function of time. The vertical line at 2.8 s indicates the median RT in the collaborate condition. Shaded areas in both panels indicate 95% percentile-bootstrap confidence intervals (1,000 bootstrap iterations). (Color figure online)

To directly assess the spatiotemporal dynamics of the coordination of search behavior, we added a temporal dimension to the overlap measure, by computing for each trial how many of the search elements had already been looked at by *both* participants, at successive points in time. This measure of cumulative overlap as a function of trial progress is shown in Fig. 7 and confirms the qualitative description of differences between conditions given above. Average overlap in the compete condition is seen to start increasing near the beginning of a trial. In contrast, the amount of overlap in the collaborate condition hardly increases until the median RT for the collaborate condition (see the vertical line in Fig. 7) is reached, and remains less than in the compete condition for the rest of the trial. This pattern was shown by the predominant part of our sample of pairs of searchers, indicating it is a robust finding. This pattern of results fits with an explanation of searchers adopting a strategy of dividing the search space in the collaborate condition, thereby avoiding looking at the elements visited by their partner until later in the trial, when most of the elements have already been looked at. Furthermore, Fig. 7 also shows that average overlap for simulated blind search steadily increased from the beginning of a trial, more rapidly than in the compete condition, specifically during the first 2 seconds. This suggests that (some) searchers do not simply ignore their competitor in the compete condition. Instead, some searchers may have adopted a strategy that involved looking at fresh tiles that had not yet been looked at by their competitor.

### Search behavior when competing

**Fig. 8:**
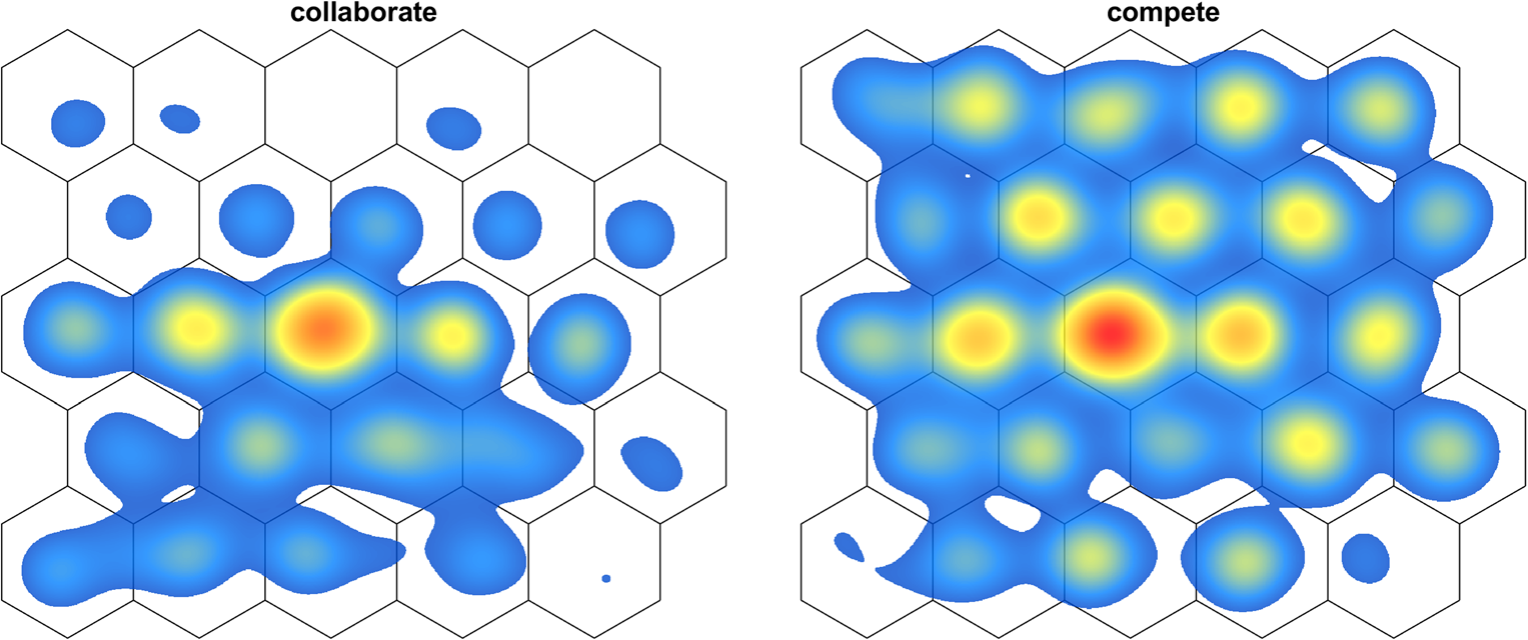
Heat maps of overlapping dwell locations for a representative example pair of searchers in the collaborate and compete conditions. (Left panel) collaborate condition; (right panel) compete condition. Panels show dwells of both searchers combined. This pair completed the collaborate condition first. Heat maps are based on number of dwells and the lowest 10% is removed from these heat maps for illustration purposes. The standard deviation of the Gaussian function used to generate these heat maps is 1.0°, corresponding to a full width at half max of 2.3°. The same color scaling is applied to both heat maps to make them directly comparable. (Color figure online)

What characterizes the search behavior of competing searchers? Figure 8 contains heat maps of an example pair of searchers in the collaborate and in the compete condition, showing the spatial distribution of overlapping dwells. Two observations stand out. First, consistent with the findings reported above, it can be seen that for, this pair, there are more overlapping dwells in the compete condition than in the collaborate condition. Secondly, it is seen that while in the collaborate condition overlapping dwells are concentrated in a central part of the search array, in the compete condition, overlapping dwells are spread across the entire search array. The streak of overlapping dwells in the collaborate condition is consistent with both participants exhibiting spatially biased distributions of dwell locations, with each looking mostly to one half of the display. The distribution of overlap in the compete condition on the other hand indicates that the dwells of both searchers were distributed much more equally across the whole search array.

**Fig. 9:**
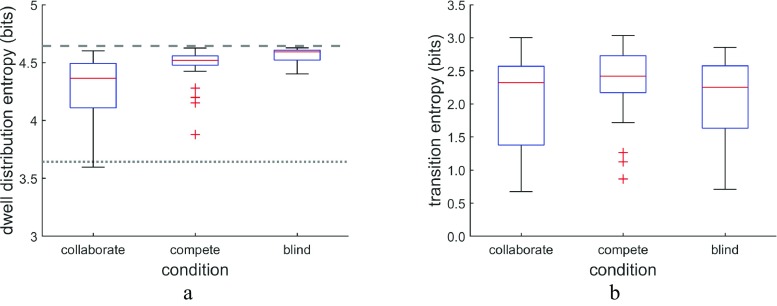
Entropy of the dwell distributions and transitions. **a** For the dwell-distribution entropy, the dashed line indicates the theoretical maximum entropy of search behavior in the 5 × 5 search array, and the stippled line indicates the expected entropy of a searcher who looked at only half the number of elements of the search array and looked at all these elements equally often. **b** For the transition entropy, the theoretical maximum value is 9.23 bits, which would reflect that all possible transitions occur equally often

To characterize looking behavior quantitatively, we computed the entropy of each participant’s dwell distribution for each of the conditions (see Fig. 9a). This showed that the median dwell-distribution entropy was significantly higher in the compete than in the collaborate condition (*Z* = 4.61, *p* < .001, *PS*_*dep*_ = 0.88), indicating that the dwell distributions in the compete condition were less biased toward a specific part of the search array than in the collaborate condition. The distribution of dwell entropy values is furthermore markedly different between the collaborate and compete conditions. Looking behavior in the collaborate condition was characterized by a wide range of entropy values, which indicates that a substantial number of participants exhibited ordered looking behavior that was predominantly directed to only a part of the elements in the search array. In the competitive condition on the other hand, almost all dwell distributions were characterized by near-maximum entropy, indicating that in this condition most participants looked close to equally often at all elements in the search array. Last, dwell-distribution entropy in the blind condition was significantly higher than in the compete condition (*Z* = 4.06, *p* < .001, *PS*_*dep*_ = 0.85). These findings regarding dwell entropy reinforce the overlap analysis presented above.

To assess whether searchers looked through the search array in a fixed pattern across trials or instead scanned unpredictably, we computed the entropy of their transitions (see Fig. 9b). We found that the transition entropy was significantly higher in the compete than in both the collaborate condition (*Z* = 3.86, *p* = .0001, *PS*_*dep*_ = 0.76) and the blind condition (*Z* = 3.22, *p* = .0013, *PS*_*dep*_ = 0.82). The transition entropy was not different between the collaborate and the blind conditions (*Z* = 0.54, *p* = .59, *PS*_*dep*_ = 0.50). Again, the range of entropy values describing the scanning behavior of the searchers was also much larger in the collaborate (and the blind) conditions than in the compete condition. The lower entropy in the collaborate and blind conditions indicates that participants searched in a more fixed and predictable pattern across trials. In contrast, the higher entropy in the competition condition indicates that search behavior in this condition was more random from one trial to the next, possibly reflecting increased interaction between searchers, which may, for instance, have yielded gaze patterns that were less predictable for the opponent.

### Questionnaire data

**Fig. 10:**
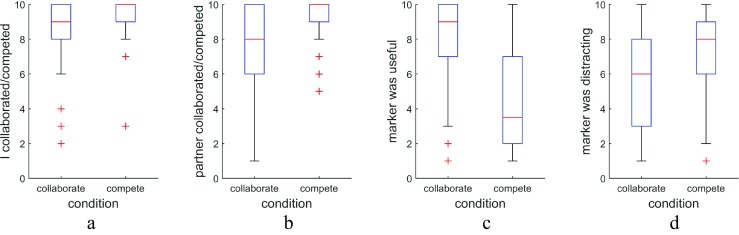
Questionnaire data, on a scale of 1 (*disagree*) to 10 (*agree*). The participants gave ratings to the following aspects for both the collaborate and the compete condition. **a** I collaborated/competed with the other person. **b** The other person collaborated/competed with me. **c**) It was useful to me to have the marker showing where the other person was looking. **d** The marker was distracting me

Participants were asked to complete a brief questionnaire at the conclusion of the experiment. This questionnaire asked questions intended to gain insight into how participants experienced working together with or competing against a partner and using the shared gaze communication channel to do so. The questionnaire contained eight items that were rated on a Likert scale (see Fig. 10).

While almost all participants indicated that they followed instructions and collaborated with or competed against their partner, they scored their partner as collaborating significantly less well than they rated themselves (*Z* = 2.53, *p* = .011, *PS*_*dep*_= 0.35). There was no difference in perceived competitiveness rating given to oneself and to the partner (*Z* = 0.31, *p* = .76, *PS*_*dep*_ = 0.18). Furthermore, the gaze marker was rated as significantly more useful (*Z* = 3.72, *p* = .0002, *PS*_*dep*_ = 0.74) and significantly less distracting (*Z* = 3.56, *p* = .00037, *PS*_*dep*_ = 0.82) in the collaborate than in the compete condition.

**Fig. 11:**
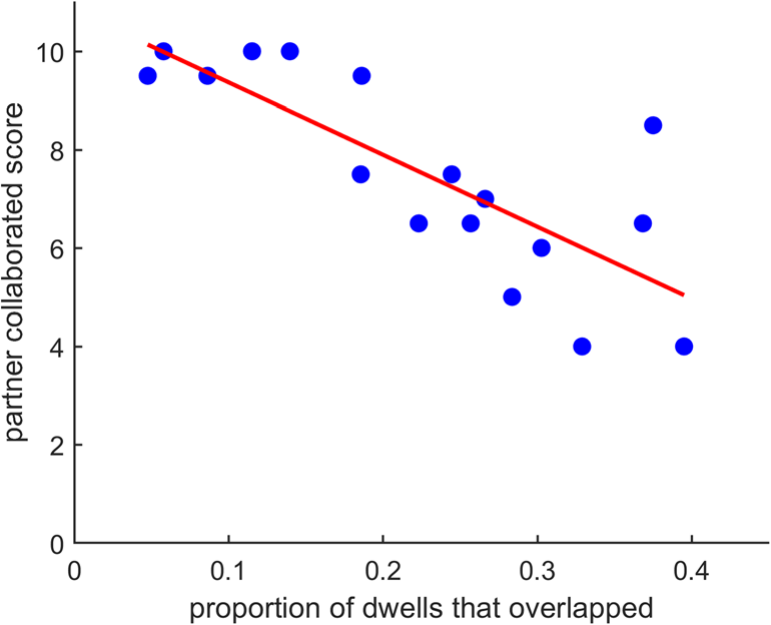
Judgments of collaboration quality as a function of overlap. Scatterplot of the proportion of dwells that landed on a hexagon that had previously been looked at by the other searcher versus the collaboration rating given, along with best-fit regression line

Above, we have used overlap in inspected search elements, or rather a lack of it, as an indication of successful collaboration. To further examine how overlap is related to subjective ratings of the collaboration and to validate the notion that less overlap indicates successful collaboration, we asked whether pairs who rate their collaboration higher also show less overlap in the collaborate condition. As seen in Fig. 11, a pair’s overlap was strongly correlated with their collaboration ratings (*r* = −0.79, *t*(15)= −4.96, *p* = .0002), such that less overlap was related to higher ratings.

## Discussion

In this study, we examined how pairs of participants searched in a shared visual space when provided with real-time information about where their partner was looking. The pairs of searchers were instructed to either collaborate with each other or compete, and we examined how the spatiotemporal coordination of search behavior of the searchers differed between these two instructions. Unless noted otherwise, all comparisons of performance and behavior of search pairs discussed below are relative to a baseline blind condition that simulated the expected performance of two searchers who could not see where the other was looking.

### Collaboration

When examining search performance, we found that showing the paired searcher’s gaze position led to significantly shorter search times. Compared with the individual condition, median search time for the pair was nearly halved in the collaborate condition, indicating close to ideal collaboration, which translated to a 1.8-times increase in the number of targets found per second. Importantly, the speed-up enabled by coordinating search behavior did not come at the cost of accuracy in the collaborate condition, as error rates remained the same as when searching alone. This suggests that having an indicator on the screen showing where a partner is looking did not have any negative effect on search performance.

What were the spatiotemporal dynamics of collaboration in our experiment? This is best understood by means of a comparison between search behavior in the collaborate and the compete conditions. First, we found that less overlap in visited elements occurred in the collaborate condition than in the compete condition. Second, this finding of reduced overlap was supported by an analysis of how overlap unfolds during a search trial. We found that while in the compete condition there was an initially slow but nonetheless steady increase in overlap over time, overlap in the collaborate condition hardly increased until the median reaction time was reached. These findings together indicate that search behavior in the collaborate condition is characterized by avoiding overlap, a strategy which resulted in a dividing of the search space.

We furthermore found that ratings of how well the partner searcher collaborated were strongly related to the amount of overlap in a pair’s dwells—that is, search pairs with little overlap in fixated hexagons also rated the collaboration higher. That we found a relationship between overlap in elements looked at and the collaboration rating strengthens the notion that the amount of overlap between searchers is a proxy for collaborative behavior, and it also suggests that searchers had an intuitive understanding that overlap would be counterproductive to efficiently (fast and accurately) completing the search as a team.

An efficient way to search collaboratively and avoid overlap with the partner in elements looked at is to look in a fixed and predictable pattern throughout all trials. In this case, both searchers could, but do not have to monitor each other or otherwise interact, as they know what to expect from each other. This prediction was borne out by our data. Both the dwell-distribution entropy and the transition entropy were lower in the collaborate than in the compete condition, further confirming that collaborative search was more biased to proceed over a subset of the elements in the search array and that this search was executed using more fixed and predictable looking patterns. The more predictable looking pattern when collaborating may reduce the need for searchers to monitor each other’s search behavior to avoid overlap. Nonetheless, we cannot determine whether searchers monitor each other throughout the block of trials or not because it is not necessary to fulfill the task assigned in this experiment.

Brennan et al. (2008) have shown, and we replicate here, that collaborating searchers adopted a division-of-labor strategy. Brennan et al. (2008) state that this strategy enabled “collaborating searchers [to avoid] redundant effort by segregating their gaze in space” (p. 1473) through “[agreeing] on who should search where [and] establish[ing] a virtual boundary demarcating these regions” (p. 1467). Searchers thereby “divided the display, each searching roughly half of the items” (p. 1470). According to Brennan et al. (2008), searchers who implemented this strategy “coordinated their fixations dynamically” (p. 1473) through being “peripherally aware of where their partner was looking” (p. 1473), thereby “allowing a more flexible and dynamic division of labor” (p. 1475). However, it should be noted that the results of Brennan et al. (2008) cannot exclude a second collaboration strategy. Using this alternative strategy, searchers would not explicitly aim to establish a division of space, but instead behave according to two principles: (1) start at opposite ends of the search array and (2) work toward each other one horizontal or vertical sweep at a time or in some other methodical manner. Following these behavioral rules, the two searchers would meet in a location in the search array that is determined by their relative search rates. If the searchers continue in the same manner when they meet, they would naturally continue into their partner’s side, in effect checking if their partner missed the target. This strategy would naturally lead to a separated distribution of fixations across the search array that is shaped according to the searchers’ relative ability without the need to negotiate the boundaries of a search area. It is also important to note that successfully implementing this strategy does not require real-time interaction between the searchers as Brennan et al. (2008) propose takes place during collaboration. This is because as soon as the two searchers adopt fixed searching patterns that start at opposite sides of the search array, each searcher could ignore what their partner does while still accomplishing an efficient division of the search space and naturally checking on each other when one has made a mistake.

It should be noted, however, that the data of the current experiment is not suited for distinguishing which of these two strategies best describes searcher behavior in the collaboration condition, as our results are compatible with both strategies. While we do find that scan patterns are more similar and predictable in the collaborate condition, as expected when following our strategy and, strictly speaking, not required by Brennan et al.’s (2008) strategy, the predictability of the scan patterns in the collaborate conditions did not differ from the blind condition, thus yielding no clear support for the proposed strategy. Future research should examine this issue further, possibly by manipulating at what time during a trial or during a block the gaze marker indicating where the paired searcher looks is visible.

### Competition

What did scan patterns look like when competing? We hypothesized that scan patterns would be less consistent across trials as searchers may interact more with each other in the competition condition than in the collaborative condition. For example, more unpredictable search behavior may be observed in the competition condition. This may be advantageous because being predictable could increase the competitor’s ability to remain one element ahead, thereby increasing their chance of winning as they would be the first to visit more of the elements in the search array. We used the dwell-distribution entropy and transition entropy measures to examine whether looking behavior was less ordered (more random and unpredictable) in the compete condition that in the collaborate condition. First, the dwell-distribution entropy indicated that dwells in the compete condition were less biased toward a specific subset of the search array and instead more equally spread across all elements in the array than in the collaborate condition. Second, the transition entropy indicated that scan patterns were more variable in the compete condition, as compared with the collaborate condition or when searching alone. We conclude that, when competing, searchers interact more with each other and therefore exhibit more variable and less predictable scanning behavior, both in terms of dwell location and in the order at which they look at search elements. We propose that such attunement to the competitor’s search behavior is adaptive for competitive search.

Overlap was lower in the compete condition than in the blind condition, especially early in the trial. This is possibly an efficient strategy, as it is less likely that a target is found at locations in the array where the competitor has already looked than at locations that have not yet been inspected by either searcher. However, if such a strategy was employed, it was not followed to an extent that yielded equally little overlap as in the collaboration condition. It is possible that more overlap was found in the compete than in the collaborate condition because it is harder to avoid looking at the same elements as a competitor who employs the unpredictable scanning patterns seen in the compete condition than the more predictable patterns observed in the collaborate condition.

Last, when we instructed participants to compete, we find that search sped up compared with the collaborate condition, but that error rates also increased. The increased rate at which participants sampled the search array in this condition compared with the collaborate condition is possibly due to increased pressure to be faster than the opponent. The increase in errors may reflect that the number of elements inspected per second was higher in this condition than is ideal for accurate identification of the search elements. Nevertheless, this decreased search time and increased error rate did not lead to a difference in the efficiency of search between the compete and collaborate conditions, as indicated by the equal number of targets found by a pair of searchers per second in both conditions.

### Implications of findings

The present findings suggest that both collaborative and competitive team search setups can enable more targets to be found in the same amount of time than single searchers working alone. Multiperson visual search using shared gaze may thus be an interesting method for applied settings such as airport security checks, product quality control, radiology, and pathology. An important question, then, is, how to get the best performance out of a team of searchers? Should they be incentivized to collaborate, or to compete? In our setting, we found no difference in the effectiveness of search between the two instructions, since the same number of targets were found per second. Nonetheless, the instruction to compete led to an increase in error rate and as such may not be suitable for tasks where mistakes are costly. In general, which type of interaction between searchers should be incentivized depends on the relative importance of accuracy and speed for the task.

In many applied search settings, such as rejecting faulty products from a production line, an increase in search efficiency directly translates to an increase in production volume as a larger number of products can be inspected in the same amount of time. Compared to an individual performing the search task alone, our results have shown that there are multiple ways to increase total search effectiveness by adding a second searcher. The simplest option is to add a second searcher, but not invest in the technical solutions required for the team to coordinate their search. Based on our results, this would allow the production line to output a 1.5-times larger volume in the same amount of time. Enabling the searchers to coordinate their search and avoid double work, as we have done in the collaborate and compete conditions of this experiment, allows for an additional 1.2-times increase in product volume over uncoordinated team search, or an 1.8-times increase over a single searcher.

A second option for coordinated team search would be for a certain division of labor to be imposed on the searchers in space (assigned sectors) or time (search arrays are parallelized over the searchers). Given that overlap in inspected elements remained significant in the collaborate condition (a median of 23% of dwells landed on hexagons that had already been looked at by the other searcher) and that adding a second searcher did not yield a doubling of search effectiveness, it is probably possible to use such imposed divisions of labor to make team search still more efficient. To optimize team performance, the division of labor could be adapted to the relative abilities of the searchers so that the faster searcher has more elements or screens to search. The downside of such imposed divisions would be that it lacks incentive to, or makes it impossible for searchers to, check their partner’s assigned array when they have finished scanning their own, which may prevent optimal team performance from being reached.

Task performance may be sensitive to other aspects of the collaborative search task. These factors may lie at the heart of the inconsistent results of previous studies as to whether shared gaze benefits search performance. For instance, it appears that the doubling of search speed of the pair when having two searchers collaborate through shared gaze disappears when the task requires each searcher to monitor the other closely because the searchers need to reach consensus on the target’s location (Neider et al., 2010; Yamani et al., 2017). This may, however, be due to an interface problem. Perhaps if searchers would be provided with a visual or auditory notification that their partner has found the target, then this would alleviate the increased need to monitor where the partner is searching during this type of task, enabling them to use the same beneficial division of space strategy as we report until the target has been acquired by one of the searchers. Furthermore, an interesting parallel can be drawn between the findings of these studies and the results in the compete condition in this study. That searchers in the compete condition showed more overlap and only a minimal speed-up in reaction times despite a relatively larger speed-up in item inspection rate could suggest that searchers monitored their opponents more in this condition than that they monitored their partners in the collaborate condition.

Other aspects of the interface of the task, such as the method of visualizing shared gaze, could also have an influence on the results. Instead of using a simple dot or ring to indicate current gaze position of the partner (Brennan et al., 2008; Messmer et al., 2017; Neider et al., 2010; Yamani et al., 2017), we highlighted the hexagon that is looked at by the partner. This may have contributed to the highly efficient collaborative search behavior displayed by our participants, because our highlighting method has two benefits over a dot or ring for displaying shared gaze. First, our highlighting method removes from the visualization most of the noise in the eye-tracker signal that would cause a point to jitter on the screen, as long as noise does not cause the reported gaze signal to travel outside of the viewed hexagon for more than a single sample. Second, the highlight never occludes any search element, allowing participants to search together without obstructing each other’s view of the search array.

Shared gaze has been conceptualized as simply another pointing device by Müller, Helmert, Pannasch, and Velichkovsky (2013), who, in a task where a novice solving a puzzle was provided with either a visualization of an experts’ gaze positions or their mouse cursor movements, found that shared gaze conferred a performance benefit compared to no guidance, but that showing mouse pointing movement led to even more efficient cooperation. They suggest this was because the information provided by the gaze cursor was ambiguous, because the gaze cursor showed both eye movements made with communicative intent and eye movements that were irrelevant for the task. As such, Müller et al. (2013) suggested that the novice viewing the gaze cursor may have been more cautious in acting upon it. Would we also expect an even larger speed-up of search in our task when the two searchers communicated via mouse movements? Probably not. The relative success with which collaborative tasks can be supported by visualizing gaze positions compared with mouse movements likely depends on the task, and specifically on the role that the gaze or mouse cursor fulfils in it. In the tasks of Müller et al. (2013), Neider et al. (2010), and Yamani et al. (2017), the gaze or mouse cursors were used to communicate specific locations at specific times during the task. In this case, the presence of noncommunicative eye movements may be detrimental to task performance. In contrast, in the present study and that of Brennan et al. (2008), all visualized gaze positions had the same role of indicating locations where the partner had already looked, and there were no eye movements or dwell locations that served a different role, nor any that had to be specifically attended to. Under these conditions, shared gaze is likely to be more effective than shared mouse movements, because shared gaze precisely and at high temporal resolution indicates where a partner searches, and it does so essentially for free, as shared gaze is simply a visualization of dwells made naturally during the search task. In contrast, mouse movements are intentional and slow, and making them for the partner to see would be a secondary task for the searcher that would only distract from searching for the target.

The demands placed on the viewer of a shared gaze marker also strongly depend on the task. In some cases, the marker represents a bid for joint attention and asks of the viewer to reciprocate by looking at the location indicated by the marker. Doing so is thought to establish a common reference between the viewer and the person behind the gaze marker, which is thought to be helpful for learning (e.g., Jarodzka et al., 2012; Jarodzka, van Gog, Dorr, Scheiter, & Gerjets, 2013). In other studies, the gaze marker is used to convey intentions or allow taking the perspective of another (Foulsham & Lock, 2015; Litchfield & Ball, 2011; Müller et al., 2013; van Wermeskerken, Litchfield, & van Gog, 2017; Velichkovsky, 1995) and thus requires substantial elaboration by the viewer to be used in the intended fashion. It is an open question whether interpretation of the visualized gaze positions as collaborative behavior underlies the collaboration benefits found in our study when making use of the shared gaze information, or if the visualized dwell locations are simply used as a spatial pointer that guides searchers as to where to search (cf. Cole, Skarratt, & Kuhn, 2016). While our findings suggest that the degree of collaboration exhibited by a search partner can be judged from the shared gaze marker, this does not indicate what extent of interpretation of the gaze marker is necessary to guide search behavior.

In short, we have shown that searchers can efficiently collaborate when provided with a visual marker indicating where their partner is looking, as revealed by a reaction time of the pair that was half that of an individual searcher, without an increase in error rate. When assessing the effectiveness of joint visual search in terms of the number of targets found per second, both instructions to collaborate and to compete were found to yield a 1.8-times increase over searching alone. In the collaboration condition, efficient team search was accomplished by making use of a looking strategy that resulted in a division of the search space. This spatially divided looking behavior was indicated by the lowest amount of overlap in items looked at by paired searchers throughout the trial. Furthermore, searchers from pairs that showed less overlap also rated the collaboration higher, indicating that overlap is a good indicator of collaborative behavior in a visual search task. In the competition condition, we found more overlap in dwell locations than in the collaborate condition, but analyses of the entropy of participants’ gaze behavior revealed that this came along with an increase in randomness of both dwell locations across the search array and of the scan pattern used to traverse the search array. By doing so, the competing searchers reduced the predictability of their search behavior, which we propose is beneficial to their chance of winning. Our results show that the “visual search through shared gaze” paradigm is a promising method for research in cognitive and social psychology that yields a great wealth of outcome measures reflecting different aspects of behavior, and a promising tool for enhancing performance in applied search tasks.
